# Digital Twin Platform for Water Treatment Plants Using Microservices Architecture

**DOI:** 10.3390/s24051568

**Published:** 2024-02-29

**Authors:** Carlos Rodríguez-Alonso, Iván Pena-Regueiro, Óscar García

**Affiliations:** 1 ESIT—Escuela Superior de Ingeniería y Tecnología, UNIR—International University of La Rioja, Av. de la Paz 137, 26006 Logroño, Spain; crodrigueza@ayesa.com (C.R.-A.); ivan.pena@unir.net (I.P.-R.); 2 Ayesa Ingeniería y Arquitectura, Calle Marie Curie 2, 41092 Sevilla, Spain

**Keywords:** Digital Twin, water treatment plant, artificial intelligence, HMI, microservices, edge computing

## Abstract

The effects of climate change and the rapid growth of societies often lead to water scarcity and inadequate water quality, resulting in a significant number of diseases. The digitalization of infrastructure and the use of Digital Twins are presented as alternatives for optimizing resources and the necessary infrastructure in the water cycle. This paper presents a framework for the development of a Digital Twin platform for a wastewater treatment plant, based on a microservices architecture which optimized its design for edge computing implementation. The platform aims to optimize the operation and maintenance processes of the plant’s systems, by employing machine learning techniques, process modeling and simulation, as well as leveraging the information contained in BIM models to support decision-making.

## 1. Introduction

Water is one of the fundamental resources for the development of societies. Since ancient times, the first cities developed around sources of water to advance their civilizations [1]. However, today, more than one billion people lack access to clean and safe drinking water, considered a right for all in developed countries [2]. Additionally, over a quarter of the world’s population in developing countries live in regions that will suffer from water scarcity, and the effects of climate change could further exacerbate these shortages [3]. This water scarcity is not only reflected in human dehydration but also in sanitation and hygiene issues, leading to a significant number of global health problems [4].

Sustainable Development Goals (SDGs) were framed at the United Nations General Assembly within the 2030 Agenda [5]. Among these goals are access to clean water and sanitation, aiming to optimize water treatment processes, develop and improve distribution and sanitation infrastructure, and clean up oceans, among other initiatives.

Industry 4.0, driven by the digitization of organizations and the use of the latest technologies such as big data, artificial intelligence, robotics, or the Internet of Things (IoT) [6], presents itself as an option to improve the quality of water infrastructure, reduce water losses, and enhance water quality, using these advantages to address sustainability issues in the entire water cycle [7]. This digitization can be implemented at any point in the water value chain, from resource capture to end-user consumption and return to the environment, including collection and supply infrastructure and distribution systems [8].

Among the different technologies driven by the Fourth Industrial Revolution, Digital Twins (DT) are one of the most attractive due to their process optimization capabilities [9]. In their publication, Madni et al. [10] define a Digital Twin as a virtual replica of a physical system, fed with real-time system information, enabling the incorporation of the most disruptive technologies of today, such as artificial intelligence, advanced data analytics, extended reality, or robotics, to extract value from data, automate and optimize processes, and support decision-making [11].

There is often confusion between DT and BIM modeling, so it is important to clarify that BIM models are static representations of geometric and parametric information, while DT are dynamic systems, connected to real-time information, designed to emulate the system they represent [12].

In the context of water infrastructure, Digital Twins can provide solutions such as predictive maintenance, anomaly detection, optimization of energy consumption, or improvements in sustainability, by reducing pollution and maximizing water conservation, as well as increasing benefits and reducing operating costs for public services, among others [8,13]. Additionally, these systems can also be used to enhance water resource management in reservoirs, prevent floods, improve stormwater tank management during heavy precipitation, optimize supply networks, or aid in reducing user consumption [8,14].

This paper focuses on the development of a Digital Twin platform for a water treatment plant, based on a microservices architecture. The rest of the text is organized as follows: Section 2 will address the state of the art of the technology, covering the main definitions of Digital Twins and their enabling technologies, as well as the main examples of digitization and Digital Twins in water infrastructure. Then, Section 3 depicts the main contribution of the Digital Twin platform for water treatment. Section 4 discusses the outcome of the work performed, delving into the main results of the platform and models developed and outlining the main future research directions for this research. Finally, Section 5 summarizes the key contributions and conclusions.

## 2. State of the Art

As previously mentioned, Digital Twins are virtual replicas of physical systems in continuous communication with them, transforming data into information using disruptive technologies to extract value [10]. In most cases, these Digital Twins represent a process, not just an individual system, so a Digital Twin can be considered as a system of systems [15]. In many cases, these processes can be very complex and require a large volume of data and a high level of abstraction to be properly modeled, so it will be especially important to undergo a process of decomposition and analysis to understand them correctly.

From one definition, it is concluded that the fundamental elements that make up the Digital Twin are the physical asset, the virtual system, and the communication among them [16]. This communication between the physical and virtual twins results from data acquisition processes, with the IoT (Internet of Things) being one of the main enablers of Digital Twins [17], such as in fields like agriculture and its monitorization [18]. Nevertheless, disconnected data will be of limited use, so it is the transformations performed by the Digital Twin that add value to this information. These transformations take the form of advanced data analytics, the use of artificial intelligence techniques, or behavior simulation through modeling [19,20].

**Figure 1:**
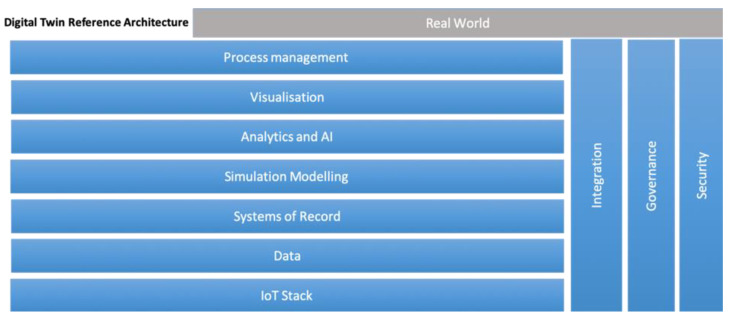
Reference architecture presented by Standfor-Clark et al. [21] for the development of Digital Twins.

To develop a Digital Twin, it is essential to understand each of the actors involved in the process. In order to establish a foundation for the development of Digital Twins, the article developed by Standfor-Clark et al. [21] presents a reference architecture for Digital Twins, consisting of seven layers that define the different functionalities of the Digital Twin and three cross-cutting layers responsible for the governance, security, and integration of Digital Twin elements (see Figure 1).

On the other hand, IBM [22] lists some of the benefits of Digital Twins, such as improvements in design and research through their use in product development, enforced by generating simulation environments and data analysis; improved process efficiency by identifying premature failures through Digital Twins or using results in new production batches; and end-of-life product assessment by evaluating which assets in the system are more deteriorated and which can be reused in other processes.

In the context of water infrastructure, Digital Twins and process digitization offer improvements for all the needs in this area. In the paper written by Banerjee et al. [14], the importance of digitization in water management is presented. In this, an evaluation of three digitization systems implemented across two different cities is carried out: Singapore, a highly developed and technologically advanced city, and Bangalore, a developing city. The digitization measures evaluated include a smart water meter system, the implementation of a SCADA device, and the development of a flood alert system.

In the case of Singapore, the implementation of these technologies aimed to improve existing infrastructure and citizen satisfaction, seeking synergies with other city systems to maximize the benefits of these improvements. Conversely, in Bangalore, the measures were taken to reduce water losses and unauthorized usage, imposing the cost burden of these measures on citizens.

The results showed that in the case of Singapore, the implementation of these systems, starting from an infrastructure with very low water losses, allowed for a reduction in water consumption by citizens, improved water quality and supply systems, and the ability to harness heavy rainfall, in coordination with city territorial management, to enhance water reserve systems in the city. In contrast, Bangalore’s focus on reducing water losses in all its measures meant that they did not consider synergies with other initiatives, resulting in very poor results that had to be subsidized by citizens, with a return on investment that did not cover the costs.

In the report presented by Hernandez Sancho [23], the benefits of digitization in natural resource sustainability management are highlighted. To demonstrate the benefits, a practical case is presented, in which, based on data from 400 pump blowers over the last 10 years, it is determined that the variables most related to failures in these blowers are operating hours, the presence of sand in wastewater, cavitation, or lower maintenance frequency than indicated by the manufacturer. These findings allow for the development of a strategy to reduce these factors whenever possible, improving process efficiency and reducing the need for corrective maintenance or equipment replacement, demonstrating the benefits of digitization and data analytics derived from this process.

In the context of Digital Twins for water infrastructure, Johnson et al. [24] presented two cases of Digital Twins in a book published by The International Water Association.

The first case focuses on a Digital Twin of the sewage network in the city of Gothenburg, with the aim of reducing wastewater discharges into the environment, resulting from heavy rains in combination with household waste. The Digital Twin consists of a model of the sewer system, using data on flows and water levels in the network, as well as at treatment plants. This Digital Twin, presented to users along with weather forecasts for the next two days, implements a network management strategy to optimize the pumping system to maintain a constant flow into treatment plants for the next 12 h.

The results presented by the Digital Twin showed a reduction of up to 50% in untreated water discharges into the environment, totaling 1.5 billion liters. It also improved process efficiency by maintaining more stable flows, reduced the risk of critical situations through predictive algorithms, and increased the capacity to address problems with pumps or at the entrance to water treatment plants through predictive control.

In the second case presented by Johnson et al. [24], a Digital Twin was developed by the public utilities of Singapore in collaboration with the company Jacobs to support decision-making in wastewater treatment plants. It was based on information collected by the SCADA system and the Laboratory Information Management System (LIMS) to develop a hybrid simulation model of plant hydraulics and control. This model allowed for a comparison between system outputs and simulated data, thus providing anomaly detection, scenario simulation to anticipate potential infrastructure modifications, and a 5-day prediction of events in the treatment plant to assist in operational decision-making.

In the context of fluid dynamics process simulation, Chiva Vicent [25] presented an alternative use of Computational Fluid Dynamics in operational decision-making for Digital Twins at a conference hosted by aguasresiduales.info. The development of this approach highlights the advantages of CFD models for calculating the evolution of processes and the hydrodynamics associated with biological reactors in wastewater treatment plants.

However, the timeframes handled by these systems are not feasible for use in operational Digital Twins that assess plant operational development, as the simulation time required exceeds the process duration. To address this challenge, Chiva Vicent proposes the use of neural networks to simulate system behavior. Trained through CFD process simulations, these neural networks can provide results with minimal error margins compared to those obtained from CFD system execution, and within seconds instead. This enables the use of these neural networks in operational Digital Twins, allowing the simulation of water treatment process outcomes and obtaining results before they occur, facilitating the optimization of plant operation and control strategies.

## 3. Contribution

As indicated in the previous sections, the main objectives pursued with the Digital Twin platform for the sewage treatment plant are as follows:

- Optimizing Operational Efficiency: By leveraging real-time sensor data and predictive analytics, the platform aims to enhance the decision-making process, ensuring that treatment operations are as efficient and effective as possible, preventing, for instance, untreated water being released to the population;
- Predictive Maintenance: Utilizing machine learning models, the platform predicts equipment failures, allowing for timely maintenance that prevents unscheduled downtimes and extends the lifespan of the plant’s machinery;
- Process Simulation: Through the ASM1 model, the platform simulates the biological treatment processes, aiding in the planning and optimization of operations to handle varying inflow conditions and maintain compliance with environmental standards;
- Resource Management: By integrating with BIM models, the platform supports the management of physical assets, helping to optimize resource allocation and reduce waste;
- Enhanced Decision Support: The platform provides a comprehensive view of the plant’s operations, enabling operators to make informed decisions rapidly and effectively.

### 3.1. Platform Architecture

This section will cover the development of an architecture for Digital Twins based on microservices deployed on a server on premise. These microservices could also be deployed on a cloud server and make use of services offered by various cloud providers, but this strategy has been followed to aid the development of the proof of concept.

The adoption of a microservices architecture yields several benefits, crucial for optimizing system performance and enhancing operational efficiency. Firstly, the modular nature of microservices facilitates greater flexibility and agility in the development and deployment of new use cases, following an agile methodology for developing the Digital Twin. By decomposing the system into smaller, loosely coupled services, updates and modifications can be implemented seamlessly without disrupting the entire ecosystem. This modularity also enables scalability, allowing individual components to scale independently, based on specific resource demands, thereby ensuring optimal performance even under varying workloads and evolving requirements. Furthermore, the decentralized nature of microservices promotes fault isolation, preventing issues in one service from affecting others and enhancing system reliability and resilience. Additionally, the use of microservices fosters interoperability, by enabling better integration with external systems, data sources, and services, thereby facilitating the exchange of information and enhancing the overall functionality of the Digital Twin.

To achieve this, the architecture starts with the development of a central gateway and control service, named the API Gateway, which acts as the orchestrator of the microservices and serves as the link to the user interface application [26].

To enable communication between legacy systems such as SCADA and the API Gateway, this architecture proposes the use of OPC UA, as suggested by Hoffman et al. [27]. OPC UA is a unified communication standard for information modeling and real-time data exchange [28]. Using these protocols allows the development of Application Programming Interfaces (APIs) to access data from OPC UA sensors. These APIs, combined with the use of gateways, enable operational data to be brought to the network for use by the Digital Twin [20].

The communication service will leverage the APIs exposed by the OPC publisher to obtain system data and send it to the gateway. The gateway then sends a request to the data ingestion service to store the obtained information. Prior to this data publication, it will be necessary to verify the identity of the device sending the data to the platform. This task will be performed by the authentication service.

**Figure 2:**
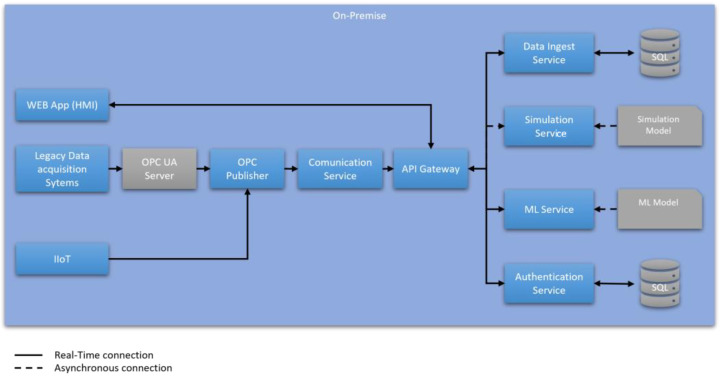
Simplified architecture of the Digital Twin platform.

Once the data is published, the gateway service will coordinate the collection of necessary data and send it to the analytics service, based on machine learning in this case, to obtain the analysis results and store them again through the data ingestion service, as Figure 2 shows.

Finally, this simplified architecture proposes the development of a web application as users’ interface. These users will identify themselves on the web page by sending a request to the authentication microservice through the gateway, granting them access to the platform if their identity is confirmed.

To ensure user security and privacy, as this is a sensitive infrastructure, the data will be encrypted in the users’ database, following a salt and hash strategy [29]. The authentication system will return a temporary token, which will be used by the API Gateway before calling any microservice, ensuring route security and maintaining session temporality. This API Gateway will filter all communications with the various microservices, verify the identity of users using a username and password against an encrypted database, employing hash techniques strengthened with the use of salts to prevent decryption. Once the user is identified, the web application will provide access to a Digital Twin selection platform. Upon this selection, a digital model based on BIM models and using Autodesk Platform Service [30] will be presented, allowing access to all the data stored in the BIM model and the development of dashboards based on this data, as a product of the infrastructure digitization process. Additionally, to ensure the identity of data acquisition devices, the gateway service will also verify a token against its identity using the authentication service, allowing for the processing and storage of data sent only if the operation is validated. Also, all microservices will have been protected with firewall rules, allowing only calls from inside the server, where the API Gateway will be the only service able to call the microservices.

In addition to the functionalities developed thanks to the processing of data from BIM models, this platform will allow the control of data and use cases presented by the Digital Twin. The gateway service will request the latest stored data from the data ingestion services. Upon selecting one of the monitored assets on the platform, it will display the latest data from the data acquisition devices in a graph, as well as the results of data analysis using the simulation and machine learning services employed. Furthermore, this platform also allows the launching of online simulations, obtaining results to support decision-making.

In designing the Digital Twin platform, it was opted to pursue edge computing implementation due to the following considerations and tools [31,32]:

- Real-time Data Processing: The platform was tailored to process sensor data on-site at the water treatment facility, enabling immediate response to changing conditions, without latency;
- Efficient Data Handling: To manage bandwidth, the system was optimized to preprocess data at the edge, reducing the volume of information required to be sent to the cloud, if this was to be extrapolated to other facilities, and focusing on transmitting actionable insights;
- Scalable Microservices: The architecture uses microservices that can be individually scaled and updated, allowing for flexibility in expanding or upgrading the system’s capabilities at the plant level;
- Enhanced Security Measures: Recognizing the critical nature of water treatment infrastructure, edge allows for another layer of robust security protocols and reduces the amount of information shared off premises;
- Autonomous Operations: The platform can maintain operations during network disruptions (still common in remote areas), ensuring continuous water treatment processes and data synchronization once connectivity is restored.

By adopting a 5-dimensional model [33], the design of the platform is prompted to optimize operations, maintenance, and decision-making within water treatment facilities. The following analysis delves into the specifics of each dimension, tailored to the unique requirements of the water treatment industry and leveraging a microservices architecture for improved scalability, flexibility, and efficiency.

- Cyber-Physical Data Store Layer: This layer is where real-time operational data from the water treatment plant is captured and stored. Sensors and IoT devices collect data on water quality, flow rates, and equipment status. This information is essential for creating an accurate virtual representation of the plant’s physical systems and processes;
- Primary Processing Layer: The data from the first layer are processed to convert raw sensor readings into a structured format. This involves standardizing data for compatibility with the Digital Twin platform and ensuring that the data flow is maintained efficiently and securely;
- Models and Algorithms Layer: In this layer, the water treatment plant’s processes are modeled using computational algorithms. These algorithms simulate the behavior of physical assets and processes, such as filtration, chemical dosing, and sludge treatment, providing a basis for the analysis and prediction functionalities of the Digital Twin;
- Analysis Layer: Utilizing the processed data and models, this layer performs advanced analytics to predict equipment maintenance needs, optimize treatment processes, and improve plant performance. Machine learning (in the case of the water pump) and physics-based (sludge) models analyze patterns and trends, enabling predictive maintenance and operational insights;
- Visualization and User Interface Layer: The final layer is where the processed data, analytical models, and predictions are presented to the users through an interactive interface. This layer allows plant operators to visualize plant performance, receive maintenance alerts, and make informed decisions based on real-time data and predictive insights.

### 3.2. Machine Learning Service

One of the main goals while developing the Digital Twin platform for a water treatment plant is the implementation of a predictive maintenance service. This service is a crucial component that can significantly reduce downtime and maintenance costs [34]. The service developed in this framework uses machine learning techniques to predict potential failures in the plant’s equipment, specifically focusing on water pump drivers. More concretely, machine learning techniques are integrated into the Digital Twin platform to enhance the following functionalities:

- Predictive Maintenance: Machine learning models analyze historical sensor data to predict potential equipment failures, enabling proactive maintenance scheduling and reducing downtime;
- Anomaly Detection: These techniques monitor operational data for deviations from expected patterns, identifying potential issues before they escalate into serious problems;
- Process Optimizations: Machine learning assists in optimizing various treatment processes by analyzing trends and correlations in data, leading to more efficient operations being recommended as next steps;
- Data-Driven Insights: The platform utilizes machine learning to transform raw data into actionable insights, supporting informed decision-making and continuous improvement.

The predictive model aims to anticipate errors in the water pump drivers at the treatment plant within a 10 min interval, allowing for timely preventive maintenance actions. This approach is particularly valuable in water treatment plants where equipment failures can lead to untreated wastewater discharges, causing environmental pollution and potential health risks.

For gathering the data, it is key to review the data acquisition possibilities, involving precise sensor placement to ensure accurate signal capture, and feature extraction focuses on identifying parameters that are highly indicative of the system’s health. As a demonstration of the capabilities of machine learning services for the framework presented in this paper, a predictive service for errors in water pump drivers at the treatment plant was developed. This was done using a public dataset from Kaggle [35], which was labeled with normal, breakdown, or recovery operating states, with data collected every minute during the winters of 2018 and 2019. The dataset provided 52 sensor readings associated with the pump’s operational state, such as temperature, pressure, flow rates, and vibration levels.

First, data cleaning and preprocessing were performed by removing variables that had more than 1% of their values missing in the dataset and filling in missing values for the remaining variables based on the last non-null value, since the dataset was structured as a time series [36].

**Figure 3:**
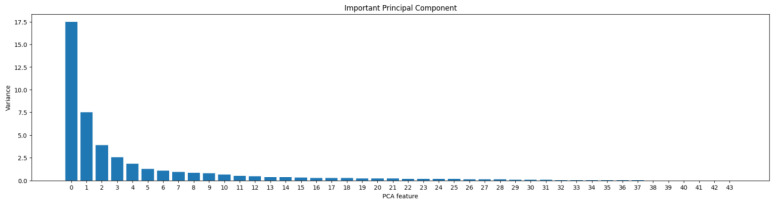
Variance of the principal components extracted from the dataset.

Given the high number of variables and their interrelationships, dimensionality reduction techniques were deemed necessary to aid in model training. The correlation matrix visualization allowed the identification of many variables that were related to each other [37], making it possible to select the most important ones and discard the rest. Nevertheless, a Principal Component Analysis (PCA) algorithm was used to reduce dimensionality, by choosing which variables of the project are relevant to be used in training the system [38], whilst keeping the most significant information from the dataset’s features, revealing the variance contribution of each. Upon analyzing the results, it was observed that the most significant variance was encapsulated within the first 10 components, with the variance diminishing progressively, following a pattern resembling a negative exponential curve (see Figure 3).

Additionally, to facilitate the model training, mitigating the influence of variables with larger magnitudes and to improve training speed, a normalization technique was applied using a standard scaler, where each sample’s values were centered by subtracting the variable’s mean and dividing by the standard deviation, resulting in a dataset in which all variables have a mean of 0 and a standard deviation of 1 [39], and therefore ensuring that each feature contributed equally to the model’s predictions.

Based on the variance results obtained from the principal components, it was decided to keep a system with the first 15 components, as it is estimated that these would be sufficient to represent the dataset.

**Figure 4:**
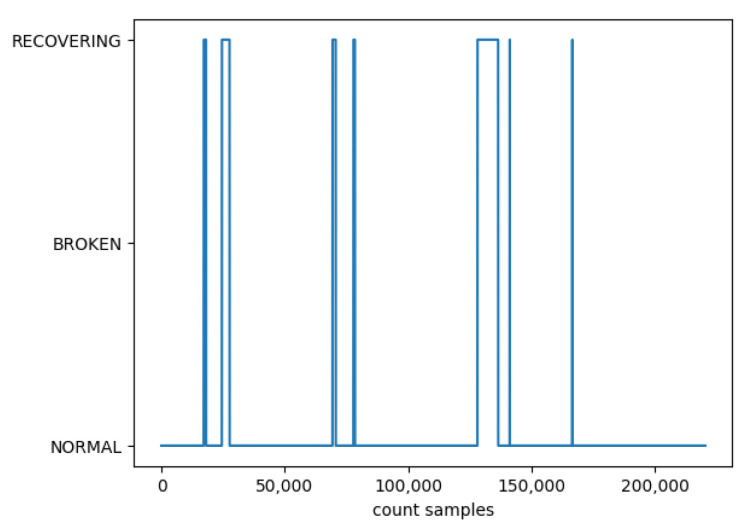
States distribution on the dataset.

Before starting with the training, the distribution of samples in the dataset was analyzed, showing that cases of normal operation are much more frequent than breakdown and recovery cases, and that all recovery cases follow a breakdown. Therefore, it was decided to identify breakdown and recovery cases in the same way, the model’s purpose being to anticipate breakdown situations, and predicting either a breakdown or recovery in the future serves the same purpose of anticipation. This is shown in Figure 4, where the transition from ‘normal’ to ‘broken’ was always followed by a ‘recovering’ and a return to ‘normal’.

The machine learning model’s objective is to predict the pump’s state 10 time intervals ahead based on the data from the last 10 time intervals. Given the need for future prediction, past data, and considering that system failures are in many cases often preceded by inefficient operation in previous periods, a recurrent neural network was employed [40], or more concretely, a Long Short-Term Memory (LSTM) Recurrent Neural Network (RNN). Among the various recurrent neural network algorithms, Long-Short Term Memory cells were used in this case, which allow for long-term memory, taking previous conditions into account in future predictions. Following the LSTM layer, a dense neural network layer was used for classification [41].

For model training, binary cross-entropy (1) was used as the cost function, which heavily penalizes incorrect classifications, ensuring that the training considers breakdown cases despite their lower frequency compared to normal behavior cases.

(1) $$F_{cost}\left(q\right)=-\frac{1}{N}\sum_{i=1}^{N}y_{i}*\log\left(p\left(y_{i}\right)\right)+\left(1-y_{i}\right)*\log(1-p\left(y_{i}\right))$$

The architecture of the neural network was selected so it is able to capture the temporal dependencies and patterns in the sensor data. These are characteristic of time series data commonly found in predictive maintenance scenarios. LSTM has the ability to learn from data points that are far apart in time, making them capable of recognizing patterns over longer sequences, which is essential when predicting equipment failures that may be based on prolonged periods of operation.

The Gate mechanism also allows the network to decide which information should be kept or discarded at each step in the sequence. This selective memory process is critical for predictive maintenance, where not all sensor readings may be equally relevant for predicting a future failure.

As seen in Figure 4, the time between normal operation and potential failure can vary significantly. LSTMs are robust to changes in the length of input sequences, meaning they can adapt to different durations of operational cycles without the need for re-engineering the network structure.

LSTMs can recognize and learn the cyclical behavior or trends present in sensor data, to anticipate future equipment states. Finally, LSTMs are also efficient in the need for feature engineering, as they can make predictions based on raw time series data.

The network architecture consisted of an initial layer of LSTM neurons, with a total of 250 neurons using hyperbolic tangent as the activation function and sigmoid activation for recurrent neurons, followed by a dropout layer. The next layer was another LSTM with the same activation functions and a total of 100 units, followed again by a dropout layer. Then, a densely connected layer with 10 units using the Rectified Linear activation function was presented, followed by a dropout layer. Finally, the output layer consisted of a single densely connected neuron with the sigmoid activation function. Since the model was a binary classification model, the output could be understood as the probability of the pump being in normal operation.

Upon training this model and evaluating the results on the test dataset, a recall of 0.977, precision of 0.984, and F1 Score of 0.981 were achieved, demonstrating the network’s ability to detect both normal operating states and breakdown states. The confusion matrix shows all the results obtained in the evaluation leading to these metrics.

**Figure 5:**
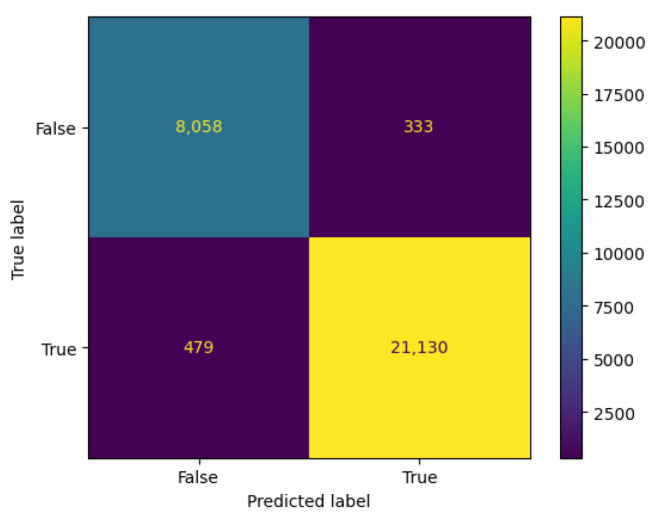
Confusion matrix of the results obtained by the predictive state model in the water pump on the test dataset.

Upon examination of the test set, it was noted that of the samples labeled as either breakdown or repair—which are critical states for predictive maintenance—only 333 were incorrectly classified as normal operation, constituting roughly 4% of the total sample size for malfunctioning states. Conversely, when considering samples labeled as normal operation, the model misclassified a mere 479 as potential breakdown or repair conditions from a total of 21,609 cases, amounting to less than 2.1% of the normal functioning sample pool. These figures underscore the model’s high accuracy in distinguishing between normal and malfunctioning operational states, validating its efficacy for maintenance applications (see Figure 5).

Using the previously trained models, this framework proposed the development of a predictive maintenance microservice. This services, based on REST API, received a request from the API Gateway service with the last data as a body request. Then, the service used the machine learning model for status prediction and returned the estimated state.

To develop predictive maintenance microservice in this research, the flask Python 3.10.12 framework was used, as per [42]. Based on this framework, an API REST access point was exposed to the API Gateway service, allowing this service to request prediction on the water pump status.

### 3.3. Simulation Model: Active Sludge Model 1

Within the framework developed in this paper, the development of an active sludge model for the reactor of the water treatment plant was presented as an example of a simulation model. This physics-based model, based on the work by Henze et al. [43], simplifies the processes carried out in the biological reactor, considering the most representative kinetic and stoichiometric processes occurring in the reactor.

In the treatment of water in the biological reactor through an activated sludge process, the water flow to be treated enters a tank where sludge or biomass, composed of microorganisms, is present to remove contaminants from the water [44]. This tank may consist of anoxic zones, where transformation occurs in the absence of oxygen, and an aerobic zone, where oxygen is consumed. For the development of this model, the variables involved in the reaction were simplified based on the decomposition of the matter involved in the processes, as indicated in Table 1.

The main reactions that occur in the tank, and which are included in the ASM1 (Active Sludge Model 1) according to Henze et al. [43], are as follows:

- Aerobic growth of heterotrophic bacteria;
- Anoxic growth of heterotrophic bacteria;
- Aerobic growth of autotrophic bacteria;
- Decomposition of autotrophic biomass;
- Decomposition of heterotrophic biomass;
- Ammonification of soluble organic nitrogen;
- Hydrolysis of trapped organic substances;
- Hydrolysis of trapped nitrogen.

Henze et al. [43] presented the relationship between components and these processes through a Peterson matrix, defining the coefficients of the relationships using stoichiometric parameters and each of the reactions using the kinetic parameters of the system.

To develop the simulation model, the equations presented in the Peterson matrix were used, implementing the equations governing the processes involved in a Simulink model. For this, a biological reactor consisting of two tanks was considered: a first anoxic tank that served the denitrification function, and a second aerobic tank, where the oxidation and nitrification process took place, followed by an ideal clarifier where no chemical transformation occurred, controlling the age of the sludge through the waste flow rate, which went to the sludge chain.

The outputs of the simulation model were the concentrations of each of the components at the outlet of the biological reactor, with special control over the total suspended solids, chemical oxygen demand, total nitrogen, and dissolved oxygen concentration. The inputs to the model were the concentrations of the components in the influent and in each of the tanks, as well as the values of stoichiometric and kinetic parameters.

**Figure 6:**
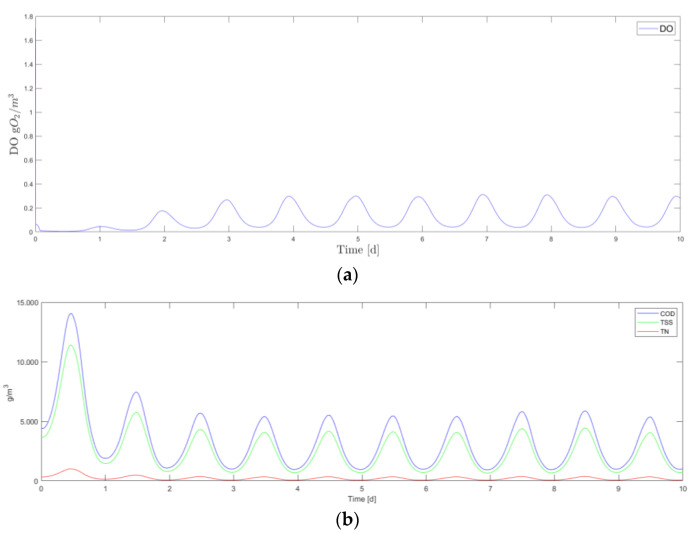
Results of the simulation using ASM1. (**a**) Dissolved Oxygent (DO) evolution on 10 days simulation. (**b**) Chemical Oxygent Demand (COD), Total Suspended Solids (TSS), and Total Nitrogen (TN) evolution on 10 days simulation.

Starting from the condition presented by Talib [45] and conducting a simulation for a total of 10 days with a sinusoidal flow rate as input with Gaussian noise, the results obtained are shown in Figure 6.

To use this biological reactor simulation service, an asynchronous strategy was followed in this article, allowing the platform to export the variables of the real system as a .csv file, also being the model fed from this file to execute the simulation.

### 3.4. Digital Twin Platform

The Digital Twin platform serves as the central axis of the Digital Twin, acting as the interface for users. Developed as a web application using .Net Core, it utilizes the previously described services to facilitate the defined use cases and features its own database to store necessary information.

The Digital Twin platform works as an access point for users of the Digital Twin of the water treatment plant. In this framework, the development of this platform was proposed as a web application, following a model-view-controller (MVC) scheme, a well-established design pattern that promotes efficient code organization and separation of concerns.

Upon initial access, this platform provided a user identification view, which operated through the authentication microservices managed by the API Gateway. The response token is stored in the session for subsequent API calls, ensuring a seamless and secure user experience. Once access to the platform was authorized, users were presented with a list of the Digital Twins they had access to, which was defined based on their user, role, or organization.

Subsequently, the platform allowed users to a view of available Digital Twins, enabling them to select the specific Twin they wish to access. This feature effectively centralizes the management of multiple Digital Twins within a single platform. To aid user navigation through the list of Digital Twins, the platform incorporates a search function that swiftly locates the desired Digital Twin. After selecting the specific Digital Twin, the BIM model resulting from the digitization process was launched whenever it was available. Therefore, these BIM models serve as a critical decision-support tool within the Digital Twin platform, providing a detailed and interactive representation of the plant’s physical and functional characteristics to inform and enhance operational strategies.

The BIM model was stored in a common data environment. In this framework, access to BIM models and their visualization was developed using the APIs provided by Autodesk Platform Services [30], but other frameworks like IFC.js [46] could also be used. The use of these data visualization tools and associated information allowed the development of functionalities that leveraged the data and provided information about each of the model’s components, such as conducting a carbon footprint analysis of each model component [47].

The BIM model is responsible for ensuring that the interface is approachable and straightforward for operators and decision-makers alike. Emphasizing a hassle-free user experience, the platform is equipped with an intuitive dashboard that presents BIM models in a navigable format, familiar to those with experience in authoring tools.

At this first stage there are few use cases, but in potential future releases, with more applications added, the selection of use cases shall be streamlined, allowing users to access relevant scenarios and data visualizations with minimal effort. Decision-making is supported by clear, interactive charts and graphs that translate complex data into actionable insights. Additionally, the platform includes a help feature and user guide/blueprint to assist developers in how to digitize other infrastructures.

This view also contained connected assets or Digital Twin use cases, which presented updated status information by reading data offered by data acquisition systems through the API Gateway, as well as simulations and predictions. For the first use case, based on the pump status data collected by the sensors, the platform estimates the pump’s condition within a 5 min window. This stored data is updated every 30 s and displayed in a graph, allowing users to anticipate abnormal states in the device and perform preventive maintenance to prevent equipment damage.

Furthermore, the platform allowed for on-demand simulations to evaluate possible outcomes in fictitious situations, enabling the assessment of potential system behaviors to enhance operational capabilities. Regarding the biological reactor, the Digital Twin platform will display state data for the reactor in both tanks, as well as the influent data entering the plant. This allows users to export the data for use in the simulation model, enabling them to assess whether the current plant conditions can handle the inflow, whether adjustments to the conditions could accommodate it, or whether the inflow should be diverted to a plant with greater capacity.

This platform is engineered to be adaptable, enabling integration with future advancements in the digitalization of the water treatment plant. This flexibility will accommodate the expected developments in sensor technology, data analytics, predictive algorithms, and automation processes.

Key to this adaptability is the platform’s modular architecture and API-first design. These features ensure that the platform can be continuously updated and expanded without major overhauls. New functionalities and improvements can be developed and integrated into the existing framework with minimal disruption, ensuring that the platform remains current with the latest industry trends and technological innovations. The platform’s design anticipates future technological developments in digitalization and water treatment. Its microservices architecture enables the incorporation of new functionalities and integration with advanced systems, ensuring that the platform remains relevant and effective in the evolving landscape of water management technologies.

### 3.5. Strategy for Digitization and Digital Twin Implementation

For the water treatment plant digitalization, it is recommended, similarly to other Digital Twin implementation projects, to implement a multi-stage strategy that begins with a rigorous data acquisition phase, capturing critical operational parameters through advanced sensing technology and the meticulous integration of BIM models to mirror the physical infrastructure.

Following this, the digitization phase constructs a high-fidelity virtual model that serves as the foundation for the Digital Twin platform. Emphasizing real-time data synthesis and predictive analytics, the strategy incorporates machine learning algorithms, such as LSTM networks, to forecast equipment conditions with high precision.

As showcased in this paper, this framework not only streamlines plant operations but also significantly enhances the accuracy of maintenance schedules, ultimately leading to improved plant efficiency and reduced operational costs. The section sets forth a blueprint for the integration of digital technologies into water treatment processes, showcasing the transformative potential of Digital Twins in industrial applications:

- Data Collection and Management: Establishing a comprehensive data acquisition system is the first step. This involves deploying various sensors and data loggers throughout the water treatment plant to collect real-time data on water quality, flow rates, energy consumption, and equipment status;
- Building Information Modeling (BIM): Integrating BIM allows for the creation of a detailed 3D digital representation of the water treatment infrastructure. This model serves as a central repository for all spatial and technical data, facilitating better planning, construction, and maintenance activities;
- Machine Learning and Predictive Analytics: Machine learning algorithms are used to analyze historical and real-time data to predict equipment failures, optimize treatment processes, and reduce downtime. In particular, LSTM networks are implemented for their ability to handle time-series data and make accurate predictions based on long-term operational patterns;
- Digital Twin Implementation: The creation of a Digital Twin involves synthesizing collected data and BIM models into a dynamic simulation that mirrors the physical plant. This virtual counterpart can be used for process optimization, scenario testing, and training without interrupting actual plant operations;
- Integration of IoT Technologies: The Internet of Things (IoT) plays a crucial role in interconnecting sensors, equipment, and control systems;
- Cybersecurity Measures: Ensuring the security of digital systems is a priority. The blueprint includes robust cybersecurity protocols to protect against unauthorized access and cyber threats, safeguarding sensitive operational data;
- User Interface and HMI: A user-friendly interface and Human-Machine Interface (HMI) is developed to provide plant operators with intuitive access to system insights, alerts, and controls, enhancing decision-making and operational oversight;
- Feedback Loops and Continuous Improvement: The blueprint emphasizes the importance of feedback mechanisms that allow for the continuous monitoring of system performance. These mechanisms enable ongoing improvements to the Digital Twin model and the overall digitalization strategy.

## 4. Discussion

**Figure 7:**
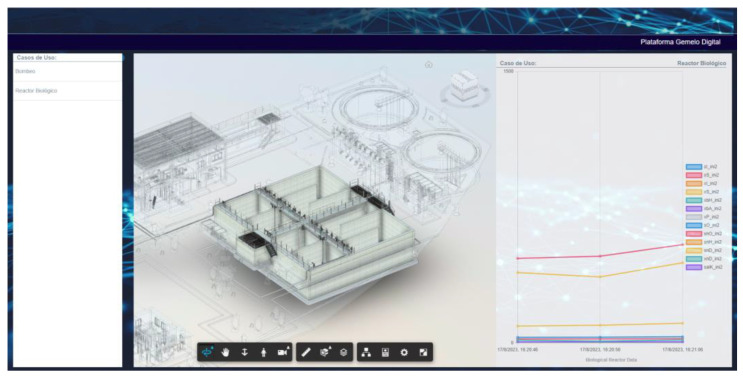
Digital Twin platform. The biological reactor and the data obtained by the data acquisition devices are displayed.

The research presented in this paper introduces a novel framework centered around the development of a Digital Twin web-based platform for a water treatment plant)see Figure 7). This platform aims to facilitate the control and management of water treatment plants by providing system information in real time and analyzing data to support decision making, on the web, giving access from any device and anywhere.

The presented microservices architecture allows for the progressive addition of new use cases for the Digital Twin, enabling better control over other system assets or creating new revenue streams. As an example, two main use cases have been presented: the first one focused on predictive maintenance using machine learning techniques, and the second centered around simulating processes within the biological reactor.

In the first use case, a predictive model for the state of water pumping pumps at a 10 min interval has been developed. Detecting a possible early failure in these systems enables preventive maintenance and the activation of auxiliary systems, thus avoiding a process shutdown that could lead to untreated wastewater discharge into the environment. Furthermore, the development of this model can be reused for other pumps, adapted to the operating conditions of each system, reducing model development times and increasing return on investment.

In the second use case, simulating the processes inside the biological reactor allows for evaluating the plant’s capabilities to handle current flow rates. This evaluation helps determine if changes in the system’s operating conditions are necessary to prevent the discharge of non-compliant flow rates into the environment.

Additionally, the platform’s integration with the Digital Twin and information analysis serves as another use case, enabling improved maintenance planning, assessment of environmental conditions, potential future design improvements, and virtual assistance for maintainers who are not physically present at the plant.

On the other hand, using the ASM1 allows the operators to monitor the evolution of the water treatment process developed in the plant. This permits the identification of potential deviations from the parameters and modifications in the chemicals concentrations of the different components involved in the process. Further development in the Digital Twin platform, such as the Digital Twin’s connection with sewage, allows for the evaluation of the treatment process in the plant for a future discharge observed in the sewerage network, and for deciding the best treatment strategy, adjusting the plant conditions to those necessary for the treatment of that discharge, or redirecting it to other plants for treatment.

From the user point of view, the Digital Twin platform features an intuitive user interface that enables operators and decision-makers to interact seamlessly with BIM models and access use cases. The design prioritizes ease of use, ensuring that even users unfamiliar with authoring tools can efficiently navigate the platform. With the inclusion of clear, chart-based visualizations for data analysis and decision-making, the platform facilitates a user-friendly experience tailored to the needs of professionals in the water treatment industry.

Future developments in this research may involve applying this framework to a real system to demonstrate its benefits, integrating the simulation model as a microservice with an exposed access interface, and implementing the system using public cloud services. Also, cybersecurity aspects may be addressed. Among them:

- Edge-Specific Security Protocols: Implementing security protocols designed for the characteristics of edge computing;
- Network Segmentation: Segmenting the network to isolate edge devices from the core network, limiting the potential impact of a security breach and containing threats within controlled segments;
- Secure Data Transmission: Utilizing encryption and secure communication channels for data transmission between edge devices and the eventually central system to prevent interception and unauthorized access;
- Regular Software Updates: Ensuring that all edge devices receive regular software updates and patches to mitigate vulnerabilities and protect against the latest security threats;
- Authentication and Authorization: Deploying strong authentication and authorization mechanisms for devices and users to verify identities and control access to sensitive data and system functionalities, therefore building on the current temporary token;
- Intrusion Detection Systems (IDS): Implementing IDS at the edge to monitor and analyze network traffic for signs of malicious activities and respond to detected threats in real-time;
- Physical Security Measures: Enhancing physical security measures for edge devices to prevent tampering, unauthorized physical access, and damage.

Furthermore, the platform’s versatility would allow for the development of new Digital Twins and their linkage, centralizing the management of regional water administrators and enhancing simulation and result retrieval capabilities as others future research. For example, integrating wastewater networks with various water treatment plants could anticipate incoming flow rates to treatment plants and simulate process outcomes, assisting in optimizing the control and distribution strategy for sewer network discharges.

## 5. Conclusions

The main conclusions of the work presented above can be summarized as follows:

- A novel microservices-based Digital Twin platform tailored for water treatment plants that integrates real-time data and BIM models;
- Utilization of LSTM neural networks for predictive maintenance to improve operational efficiency and minimize downtime;
- Simulation of the water treatment process using an Active Sludge Model, providing insights into plant operations;
- Optimization for edge computing, ensuring data efficiency and secure processing in critical infrastructure;
- A clear blueprint for Digital Twin integration, outlining a comprehensive approach for digital transformation in water treatment.

The development of a Digital Twin platform for water treatment plants offers a forward-looking solution to the water scarcity and quality challenges exacerbated by climate change and societal growth.

By leveraging a microservices architecture, this platform provides a blueprint for the digital transformation of water treatment facilities, integrating real-time data acquisition, predictive analytics, and BIM integration within a single, user-friendly interface.

The successful application of LSTM machine learning models for predictive maintenance, and the modeling of active sludge processes through physics-based modeling, exemplify the practical benefits of the platform. These include enhanced operational efficiency, reduced maintenance costs, and improved decision-making capabilities. The platform’s edge computing design further ensures real-time processing, data efficiency, and robust security measures, making it an adaptable solution for the ever-evolving digital landscape of the water industry.

The Digital Twin platform presents a significant stride toward achieving the Sustainable Development Goals related to water and sanitation. It demonstrates the potential of Industry 4.0 technologies to revolutionize water infrastructure, providing a replicable model for global implementation. As the platform continues to evolve, future research will focus on expanding its capabilities, integrating additional applications onto the Digital Twins, and refining its predictive models to ensure that water treatment processes remain sustainable, efficient, and resilient to the challenges ahead.

## Figures and Tables

**Table 1:** ASM1 Components.

Notation	Components	Units
$S_{I}$	Soluble inert organic matter	g COD/m^3^
$S_{s}$	Readily biodegradable substrate	g COD/m^3^
$X_{I}$	Particulate inert organic matter	g COD/m^3^
$X_{S}$	Slowly biodegradable substrate	g COD/m^3^
$X_{BH}$	Active heterotrophic biomass	g COD/m^3^
$X_{BA}$	Active autotrophic biomass	g COD/m^3^
$X_{P}$	Particulate product arising from biomass decay	g COD/m^3^
$S_{O}$	Oxygen	g O_2_/m^3^
$S_{NO}$	Nitrate and nitrite nitrogen	g N/m^3^
$S_{NH}$	$NH_{4}^{+}+NH_{3}$ nitrogen	g N/m^3^
$S_{ND}$	Soluble biodegradable organic nitrogen	g N/m^3^
$X_{ND}$	Particulate biodegradable organic nitrogen	g N/m^3^
$S_{ALK}$	Alkalinity	Molar units

## Data Availability

The data presented in this study could be available on request from the corresponding author. The data are not publicly available due to confidential agreements.

## References

[1] Yevjevich V. (1992). Water and civilization. Water Int..

[2] Gleick P.H. (1998). The human right to water. Water Policy.

[3] Seckler D., Barker R., Amarasinghe U. (1999). Water scarcity in the twenty-first century. Int. J. Water Resour. Dev..

[4] DeNicola E., Aburizaiza O.S., Siddique A., Khwaja H., Carpenter D.O. (2015). Climate change and water scarcity: The case of Saudi Arabia. Ann. Glob. Health.

[5] United Nations Transforming Our World: The 2030 Agenda for Sustainable Development. UN Doc. A/RES/70/1 (25 September 2015). https://sdgs.un.org/2030agenda.

[6] Gomez-Trujillo A.M., Gonzalez-Perez M.A. (2021). Digital transformation as a strategy to reach sustainability. Smart Sustain. Built Environ..

[7] Adedeji K.B., Ponnle A.A., Abu-Mahfouz A.M., Kurien A.M. (2022). towards digitalization of water supply systems for sustainable smart city development—Water 4.0. Appl. Sci..

[8] Grieveson O., Holloway T., Johnson B. (2022). A Strategic Digital Transformation for the Water Industry.

[9] Pires F., Cachada A., Barbosa J., Moreira A.P., Leitao P. Digital Twin in industry 4.0: Technologies, applications and challenges. Proceedings of the 2019 IEEE 17th International Conference on Industrial Informatics (INDIN).

[10] Madni A.M., Madni C.C., Lucero S.D. (2019). Leveraging Digital Twin technology in model-based systems engineering. Systems.

[11] Mihai S., Yaqoob M., Hung D.V., Davis W., Towakel P., Raza M., Karamanoglu M., Barn B., Shetve D., Prasad R.V. (2022). Digital Twins: A survey on enabling technologies, challenges, trends and future prospects. IEEE Commun. Surv. Tutor..

[12] Hosamo H.H., Imran A., Cardenas-Cartagena J., Svennevig P.R., Svidt K., Nielsen H.K. (2022). A review of the Digital Twin technology in the AEC-FM industry. Adv. Civ. Eng..

[13] Bentley. Water Infrastructure Digital Twins. https://www.bentley.com/wp-content/uploads/eBook-Water-Infrastructure-Digital-Twins-EN.pdf.

[14] Banerjee C., Bhaduri A., Saraswat C. (2022). Digitalization in Urban Water Governance: Case Study of Bengaluru and Singapore. Front. Environ. Sci..

[15] Grieves M., Vickers J. (2016). Origins of the Digital Twin Concept.

[16] Singh M., Srivastava R., Fuenmayor E., Kuts V., Qiao Y., Murray N., Devine D. (2022). Applications of Digital Twin across Industries: A review. Appl. Sci..

[17] Souza V., Cruz R., Silva W., Lins S., Lucena V. A Digital Twin architecture based on the industrial internet of things technologies. Proceedings of the 2019 IEEE International Conference on Consumer Electronics (ICCE).

[18] Maschler B., Braun D., Jazdi N., Weyrich M. (2021). Transfer learning as an enabler of the intelligent Digital Twin. Procedia CIRP.

[19] Hanumann T., Swamy N.V.V.S.N., Gowtham P., Sumathi R., Chinnasamy P., Kalaiarasi A. (2022). Plant Monitoring System Cum Smart Irrigation using Bolt IOT. Proceedings of the 2022 International Conference on Computer Communication and Informatics (ICCCI).

[20] Glatt M., Sinnwell C., Yi L., Donohoe S., Ravani B., Aurich J.C. (2021). Modeling and implementation of a Digital Twin of material flows based on physics simulation. J. Manuf. Syst..

[21] Standfor-Clark A., Fran-Schultz E., Harris M. What Are Digital Twins? IBM Developer. https://developer.ibm.com/articles/what-are-digital-twins/.

[22] IBM. Qué es un Gemelo Digital?. https://www.ibm.com/es-es/topics/what-is-a-digital-twin.

[23] Hernández Sancho F. (2022). Estudio Sobre el Papel de la Digitalización en la Gestión de las Infraestructuras Hídricas de la Comunitat Valenciana. Catedra de Transformación del Modelo Económico. Economía Circular en el Sector del Agua. Universidad de Valencia. https://www.uv.es/ctransmodec/PUB/2022/2022-doc06.pdf.

[24] Grievson O., Holloway T., Johnson B., Grievson O., Holloway T., Johnson B. (2022). Artificial intelligence, Digital Twins and dynamic resilience. A Strategic Digital Transformation for the Water Industry.

[25] Chiva Vicent S. Gemelos Digitales Como Herramienta para el Soporte a la Toma de Decisiones en EDAR Mediante Modelado CFD e Inteligencia Artificial. https://www.aguasresiduales.info/.

[26] Zhao J.T., Jing S.Y., Jiang L.Z. (2018). Management of API gateway based on micro-service architecture. J. Phys. Conf. Ser..

[27] Hoffmann M., Büscher C., Meisen T., Jeschke S. (2016). Continuous integration of field level production data into top-level information systems using the OPC interface standard. Procedia CIRP.

[28] OPC Fundation (2019). Unified Architecture. https://opcfoundation.org/about/opc-technologies/opc-ua/.

[29] Sriramya P., Karthika R.A. (2015). Providing password security by salted password hashing using bcrypt algorithm. ARPN J. Eng. Appl. Sci..

[30] Autodesk Platform Service. https://aps.autodesk.com/.

[31] Kluyver T., Ragan-Kelley B., Pérez F., Granger B.E., Bussonnier M., Frederic J., Kelley K., Hamrick J., Grout J., Corlay S. (2016). Jupyter Notebooks—A publishing format for reproducible computational workflows. Elpub.

[32] TensorFlow Developers (2023). TensorFlow (v2.14.0-rc0). Zenodo. https://zenodo.org/records/8256979.

[33] Bazaz S.M., Lohtander M., Varis J. (2019). 5-Dimensional Definition for a Manufacturing Digital Twin. Procedia Manuf..

[34] Natarajan S., Thangamuthu M., Gnanasekaran S., Rakkiyannan J. (2023). Digital Twin-Driven Tool Condition Monitoring for the Milling Process. Sensors.

[35] Pump Sensor Data for Predictive Maintenance. Kaggle. https://www.kaggle.com/datasets/nphantawee/pump-sensor-data.

[36] Pedregosa F., Varoquaux G., Gramfort A., Michel V., Thirion B., Grisel O., Blondel M., Prettenhofer P., Weiss R., Dubourg V. (2011). Scikit-learn: Machine learning in Python. J. Mach. Learn. Res..

[37] Hunter J.D. (2007). Matplotli b: A 2D graphics environment. Comput. Sci. Eng..

[38] Hasan B.M.S., Abdulazeez A.M. (2021). A review of principal component analysis algorithm for dimensionality reduction. J. Soft Comput. Data Min..

[39] Singh D., Singh B. (2020). Investigating the impact of data normalization on classification performance. Appl. Soft Comput..

[40] Rivas A., Fraile J.M., Chamoso P., González-Briones A., Sittón I., Corchado J.M. (2020). A predictive maintenance model using recurrent neural networks. Proceedings of the 14th International Conference on Soft Computing Models in Industrial and Environmental Applications (SOCO 2019).

[41] Rahhal J.S., Abualnadi D. IOT based predictive maintenance using LSTM RNN estimator. Proceedings of the 2020 International Conference on Electrical, Communication, and Computer Engineering (ICECCE).

[42] Aslam F.A., Mohammed H.N., Lokhande P.S. (2015). Efficient way of web development using python and flask. Int. J. Adv. Res. Comput. Sci..

[43] Henze M., Gujer W., Mino T., van Loosdrecht M. (2000). Activated Sludge Models, ASM1, ASM2, ASM2d and ASM3. Water Intell. Online.

[44] Ontiveros G.A., Ludmila A., Campanella E.A. Evaluación del Comportamiento de PPCP’s en Dos Procesos: Ludzack-Ettinger y Ludzack-Ettinger Modificado. Ingenieria Sanitaria y Ambiental. AIDIS Argentina 2010. https://ri.conicet.gov.ar/handle/11336/13447.

[45] Bin Talib A.H. Modeling and Control of Wastewater Treatment Process. Seri Iskandar. https://utpedia.utp.edu.my/id/eprint/402/.

[46] GitHub IFC.js Documentation. https://ifcjs.github.io/info/docs/introduction.

[47] Abed A., Morten A. Artelia. Simulation and Sustainability: Bringing Carbon Impact into View for Building Design. Autodesk Platform Services. https://aps.autodesk.com/customer-stories/artelia.

